# Arterial Spin Labeling Perfusion in Determining the IDH1 Status and Ki-67 Index in Brain Gliomas

**DOI:** 10.3390/diagnostics12061444

**Published:** 2022-06-12

**Authors:** Artem I. Batalov, Natalia E. Zakharova, Ivan V. Chekhonin, Eduard L. Pogosbekyan, Anna V. Sudarikova, Sergey A. Goryainov, Anna A. Shulgina, Artem Yu. Belyaev, Dmirti Yu. Usachev, Igor N. Pronin

**Affiliations:** Federal State Autonomous Institution N.N. Burdenko National Medical Research Center of Neurosurgery of the Ministry of Health of the Russian Federation, 16, 4th Tverskaya-Yamskaya St., 125047 Moscow, Russia; nzakharova@nsi.ru (N.E.Z.); ichekhonin@nsi.ru (I.V.C.); epogosbekyan@nsi.ru (E.L.P.); sgoraynov@nsi.ru (S.A.G.); ashulgina@nsi.ru (A.A.S.); belyaev@nsi.ru (A.Y.B.); dousachev@nsi.ru (D.Y.U.); pronin@nsi.ru (I.N.P.)

**Keywords:** brain gliomas, IDH1, Ki-67, ASL, PCASL

## Abstract

The aim of the study was to evaluate the relationship between tumor blood flow (TBF) measured by the pseudo-continuous arterial spin labeling (PCASL) method and IDH1 mutation status of gliomas as well as Ki-67 proliferative index. Methods. The study included 116 patients with newly diagnosed gliomas of various grades. They received no chemotherapy or radiotherapy before MRI. IDH1 status assessment was performed after tumor removal in 106 cases—48 patients were diagnosed with wildtype gliomas (Grade 1–2—6 patients, Grade 3–4—42 patients) and 58 patients were diagnosed with mutant forms of gliomas (Grade 1–2—28 patients, Grade 3–4—30 patients). In 64 cases out of 116 Ki-67 index was measured. Absolute and normalized tumor blood flow values were measured on 3D PCASL maps. Results. TBF and normalized TBF (nTBF) in wildtype gliomas were significantly higher than in IDH1-mutant gliomas (*p* < 0.001). ASL perfusion showed high values of sensitivity and specificity in the differential diagnosis of gliomas with distinct IDH1 status (for TBF: specificity 75%, sensitivity 77.6%, AUC 0.783, cutoff 80.57 mL/100 g/min, for nTBF: specificity 77.1%, sensitivity 79.3%, AUC 0.791, cutoff 4.7). TBF and nTBF in wildtype high-grade gliomas (HGG) were significantly higher than in mutant forms (*p* < 0.001). ASL perfusion showed the following values of sensitivity and specificity in the diagnosis of mutant HGG and wildtype HGG (for TBF: specificity 83.3%, sensitivity 60%, AUC 0.719, cutoff 84.18 mL/100 g/min, for nTBF: specificity 88.1%, sensitivity 60%, AUC 0.729, cutoff 4.7). There was a significant positive correlation between tumor blood flow and Ki-67 (for TBF Rs = 0.63, for nTBF Rs = 0.61). Conclusion. ASL perfusion may be an informative factor in determining the IDH1 status in brain gliomas preoperative and tumor proliferative activity.

## 1. Introduction

Gliomas represent about 30% of primary intracranial tumors and 80% of malignant brain neoplasms. Glioblastoma, one of the most devastating brain tumors, constitutes around 45% of glial tumors and is known for poor survival despite treatment advantages [1,2,3].

The neuropathological diagnosis of gliomas underwent significant transformation within the last few years. The “classical” approach to glioma grading is based predominantly on visual characteristics, such as cytological atypia, mitotic activity, presence of necrosis, or microvascular proliferation [4].

The 2016 and 2021 World Health Organization Classifications of Tumors of the Central Nervous System added crucial value to molecular and genetic tumor status [5,6].

The isocitrate dehydrogenase (IDH) genes were among the key characteristics of brain gliomas, as they determine treatment results and prognosis in diseased patients [6,7].

Mutations in IDH1 and IDH2 genes were initially identified by exome sequencing of colon cancer and glioblastoma cells [8,9]. Later, IDH mutations were identified in several other tumor types, including acute myeloid leukemia, chondrosarcoma, and intrahepatic cholangiocarcinoma [8,10,11,12,13]. These mutations are somatically acquired and affect different arginine residues of IDH1 (R132) and IDH2 (R172 or R140). In low-grade gliomas, IDH mutations are believed to play an important role in early tumorigenesis and precede other mutations [14,15,16].

Cells with mutations in IDH genes retain one wildtype allele and rarely lose heterozygosity [17,18]. Mutations in IDH genes lead to isocitrate dehydrogenase malfunction and abnormally high accumulation of D-2-hydroxyglutarate (D-2HG) [19].

In subsequent studies, IDH1 mutation was found to be a fundamentally important element in the natural course of the disease, especially in patients with glioblastomas. The median overall survival in wildtype glioblastoma patients is around 15 months, while in patients with mutant forms of glioblastomas the median overall survival is 31 months [5].

Nowadays, studies of preoperative radiological tumor genetic status assessment are highly relevant. One of the possible candidate methods is arterial spin labeling (ASL) perfusion. ASL is a method based on labeling of the blood water protons, which play the role of an endogenous contrast agent. ASL is a totally non-invasive procedure, as it does not require any enhancement. Mapping of the cerebral blood flow (CBF), one of the key perfusion features, is the quantitative result of ASL. The main ASL techniques differ in the way the radiofrequency pulse is applied and include pulsed ASL (PASL), continuous ASL (CASL), and pseudocontinous ASL (PCASL). The latter method seems to be balanced in quality and clinical applicability. There are some positive results concerning ASL in glioma grading [20,21].

Nevertheless, the available literature has a limited number of studies investigating the relationship between blood flow measured by ASL perfusion and IDH1 mutations in brain gliomas [22,23,24,25,26].

Liu et al. (2018) revealed a significant difference in blood flow between Grade 2–3 gliomas with different IDH1 statuses [23]. Yamashita et al. (2015) found higher blood flow in wildtype glioblastomas compared to mutant forms [26]. Wang et al. (2019) detected blood flow difference only in high-grade gliomas (HGG) [25]. Brendle et al. (2018) showed higher tumor blood flow values in wildtype astrocytomas (excluding oligodendrogliomas) than in mutant tumors [22]. Lu et al. (2021) did not find any difference in blood flow between IDH-mutant and wildtype tumors [24]. Across the studies the patient groups appeared to be quite heterogenic, and the obtained results seem conflicting which motivated the current study. These facts underline the research relevance.

The most important factor affecting the tumor course is the Ki-67 tumor tissue proliferative activity index.

Ki-67 antigen is a proliferation-specific non-histone nuclear protein with a relatively short half-life. It is expressed in G1, S, and G2 phases, and throughout the mitosis. Resting cells (G0) and cells in the early G1 phase lack Ki-67 antigen expression. Ki-67 index is counted as a percentage of positive nuclei. The simplicity and reliability of this method led to its wide clinical application within various fields of oncology [27,28].

Gliomas with Ki-67 above 10% were proven to behave more aggressively and to grow faster [29,30,31,32].

Works that studied the relationship between tumor blood flow measured by ASL perfusion and Ki-67 index in patients with brain gliomas showed contradictory results [33,34].

The aim of our research was to study the relationship between tumor blood flow (TBF) measured by the PCASL method and the IDH1 status of gliomas, as well as the Ki-67 index.

## 2. Materials and Methods

This is a retrospective study. The following criteria were used for patient enrollment: surgical tumor removal or biopsy in N. N. Burdenko National Medical Research Center of Neurosurgery, preoperative ASL perfusion study, IDH1 status and/or Ki-67 assessment. The histological diagnosis was determined according to 2016 WHO criteria [5]. The study enrolled 116 patients (45 men and 71 women) aged 10 to 78 years (mean age 44 ± 13 years) with newly diagnosed supratentorial glial tumors who were examined and treated from 2012 to 2018 (Table 1). In 106 out of 116 patients, IDH1 status was studied, and in 64 out of 116 patients, the Ki-67 index was determined.

After surgical treatment, a subsequent comparison of tumor genetic characteristics with blood flow was performed. This subgroup included 48 patients with wildtype gliomas, of which 6 patients were diagnosed with low-grade glioma (LGG, Grade 1–2, WHO) and 42 patients were diagnosed with HGG (Grade 3–4, WHO). In 58 patients from this subgroup, the presence of IDH1 gene mutation was detected, of which 28 patients were diagnosed with LGG and 30 patients were diagnosed with HGG (Table 1).

Of 64 patients with Ki-67 assessment, 33 patients presented with LGG and 31 with HGG.

MR exam was performed on a 3.0 T General Electric Signa HD scanner (GE Healthcare) with an 8-channel head coil. The following pulse sequences were used: native and contrast-enhanced T1 FSPGR BRAVO with an isotropic 1 × 1 × 1 mm voxel and a zero-gap (or axial non-enhanced T1 with a slice thickness of 5 mm and a gap of 1 mm as well as enhanced axial, sagittal, and coronal T1), axial T2, T2-FLAIR, DWI ASSET with a slice thickness of 5 mm and a gap of 1 mm, and 3D PCASL (pseudo-continuous arterial spin labeling).

CBF maps were obtained by processing 3D PCASL data, which were acquired with the following parameters: 3D FSE, 8-lead helical entire brain volume scan with subsequent reformation with a slice thickness of 4 mm; FOV 240 × 240 mm; matrix 128 × 128, ZIP 512; TR 4717 ms; TE 9.8 ms; NEX 3; post-marking delay (PLD) 1525 ms; pixel bandwidth 976.6 Hz/pixel. Scan duration was 4 min 30 s.

**Figure 1:**
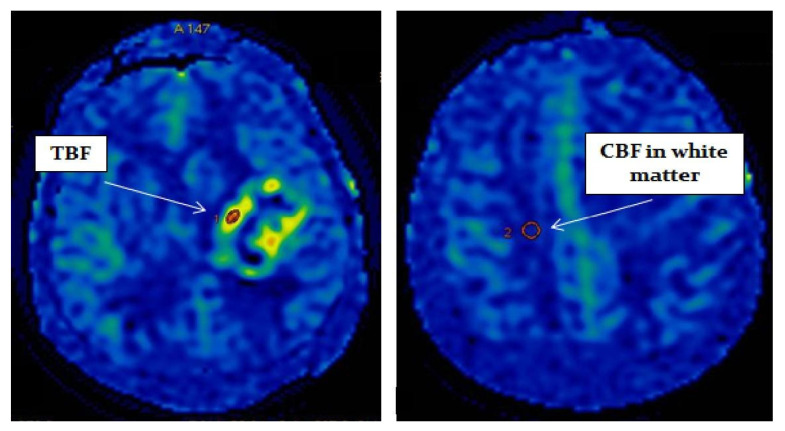
Blood flow measurement on parametric maps.

Postprocessing of the obtained data was performed using the ReadyView software package (GE Healthcare). To measure blood flow in the tumor, a region of interest (ROI) with an area of 20 mm^2^ ± 10 mm^2^ was designated in the zone with the highest CBF value (determined on color blood flow maps). In the designated ROI, the average value of TBF was calculated. To eliminate individual blood flow differences, we normalized TBF (nTBF) to blood flow in the intact white matter of the contralateral hemisphere semioval center. For this purpose, ROI with the same area (20 mm^2^ ± 10 mm^2^) as the tumor ROI was placed—Figure 1. To obtain normalized values, the obtained TBF data were divided by the blood flow in the semioval center (nTBF = maxTBF/CBF of the intact white matter of the contralateral hemisphere semioval center).

In the present study we focused on the the predictive value of maximum rather than mean TBF, as we have previously showed that maximum TBF is more informativa for glioma diagnosis than mean TBF [20].

**Figure 2:**
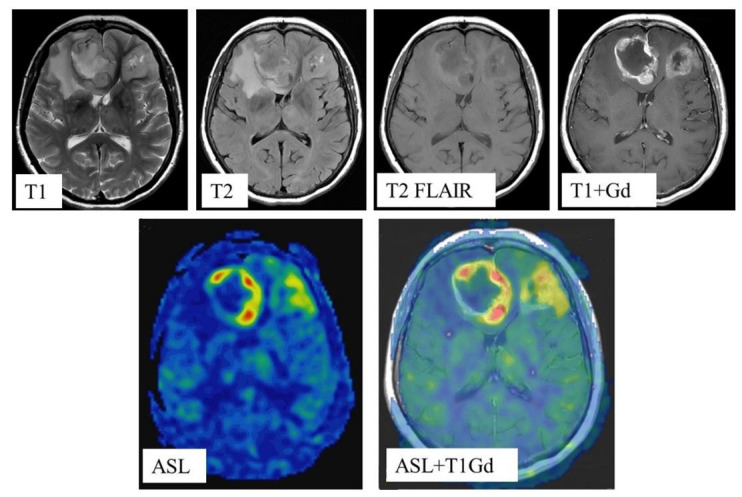
MRI scans and quantitative CBF maps of anterofrontal region IDH1-wildtype glioblastoma. ASL perfusion showed high TBF values (247.2 mL/100 g/min).

In all cases, blood flow maps were fused with anatomical images (T2, T2-FLAIR, enhanced T1) using the NeuroRegistration program (GE Healthcare)—Figure 2.

The measurements were carried out by 2 radiologists with 7- and 20-year experience (Batalov A.I., Zakharova N.E.), respectively, and the results were averaged. MRI data were anonymized, and the experts were blind to the clinical and pathological information. The coefficient of intra-observer correlation consisted of 0.87 (0.74–0.96).

Statistical processing was carried out in the R-project program (https://www.r-project.org (accessed on 5 April 2022); for ROC analysis, the pROC library was used. In this work, nonparametric methods were chosen for statistical analysis. Mann-Whitney test was performed for pairwise group comparison. Correlation coefficients were calculated using Spearman’s test.

## 3. Results

To study the relationship between TBF determined by pseudo-continuous ASL perfusion and IDH1 status, we analyzed data from 106 patients with brain gliomas. TBF and nTBF parameters in the wildtype glioma group were significantly higher compared to the IDH1-mutant glioma group (*p* < 0.001 for TBF and nTBF).

**Figure 3:**
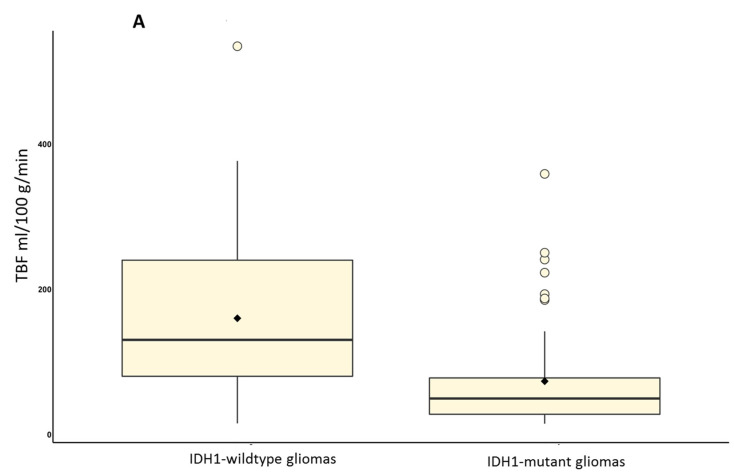

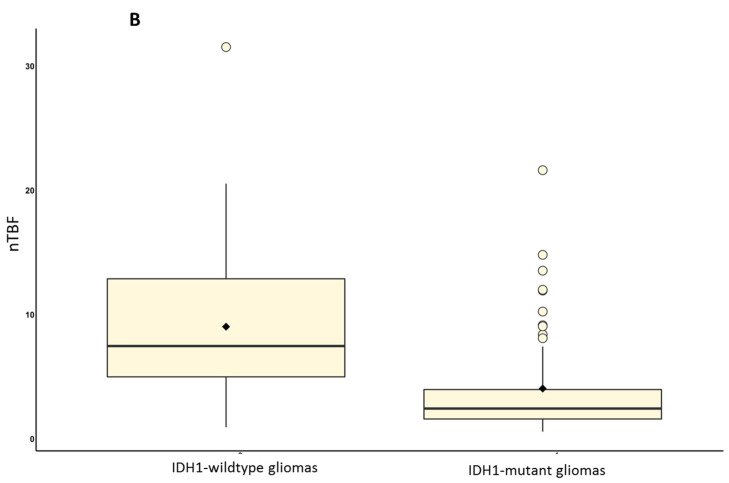
Boxplots of maximum (**A**) and maximum normalized (**B**) TBF in IDH1-wildtype and IDH1-mutant gliomas.

The maximum TBF in wildtype gliomas (*n* = 48) was 160.05 ± 109.58 mL/100 g/min, nTBF was 9.03 ± 6.17. The maximum TBF in the IDH1-mutant glioma group (*n* = 58) was 73.35 ± 70.76 mL/100 g/min, nTBF was 4.06 ± 4.14—(Figure 3).

**Figure 4:**
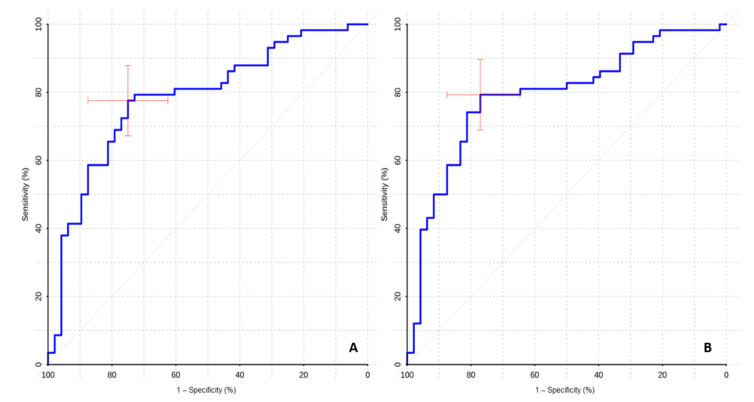
ROC curves. Comparison of maximum blood flow (**A**) and maximum normalized blood flow (**B**) between groups of wildtype and IDH1-mutant gliomas.

The ROC analysis showed high values of sensitivity and specificity of ASL perfusion in the differential diagnosis of mutant and wildtype gliomas (Table 2, Figure 4).

High AUC values in the ROC-analysis (0.783 for TBF and 0.791 for nTBF) indicate that ASL perfusion might be useful/informative in the differential diagnosis of gliomas with different IDH1 status.

In the HGG subgroup, maximum TBF and normalized blood flow values were significantly higher in wildtype gliomas compared with IDH1-mutant ones (*p* < 0.001).

The values of maximum TBF in high-grade IDH1-wildtype gliomas (*n* = 42) were 178.27 ± 104.99 mL/100 g/min, normalized values were 10.06 ± 5.91. In the subgroup of IDH1-mutant high-grade gliomas (*n* = 30), the values of maximum TBF were 110.24 ± 81.90 mL/100 g/min, normalized values were 6.13 ± 4.90.

**Figure 5:**
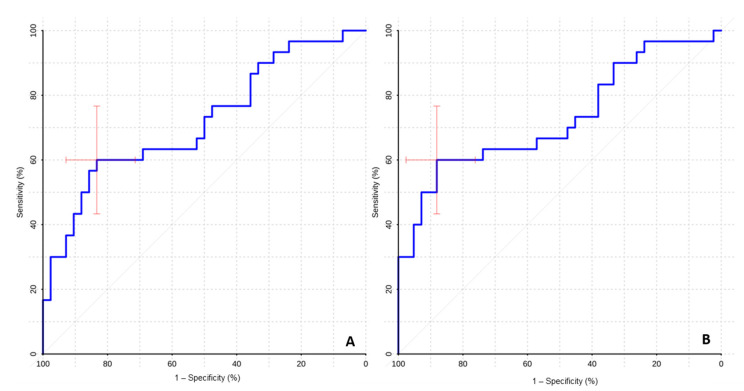
ROC curves. Comparison of maximum blood flow (**A**) and maximum normalized blood flow (**B**) between groups of malignant wildtype and IDH1-mutant gliomas.

The ROC-analysis revealed sufficient specificity and sensitivity for the differential diagnosis of malignant gliomas with different IDH1 statuses (Table 3, Figure 5).

The high AUC values (0.719 for TBF and 0.729 for nTBF) suggest ASL perfusion efficiency in the differential diagnosis of malignant gliomas with different IDH1 statuses, which is of fundamental prognostic value in this group of patients.

In the subgroup of glioblastomas, wildtype tumors (*n* = 35) showed higher values of TBF (*p* = 0.027) and nTBF (*p* = 0.022) than IDH1-mutant ones (*n* = 6). The maximum TBF in wildtype glioblastomas was 187.02 ± 106.03 mL/100 g/min, normalized blood flow was 10.58 ± 6.11. The maximum TBF in the group of IDH1-mutant glioblastomas was 95.35 ± 74.96 mL/100 g/min, the normalized blood flow was 4.95 ± 3.53. ROC analysis data are presented in Table 4.

**Figure 6:**
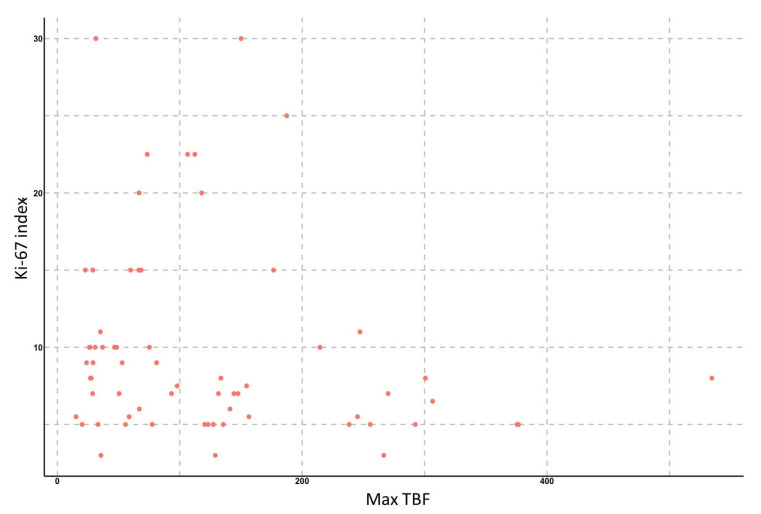
Scatterplot demonstrating the relationship between maximum TBF in gliomas of various grades and Ki-67 index.

**Figure 7:**
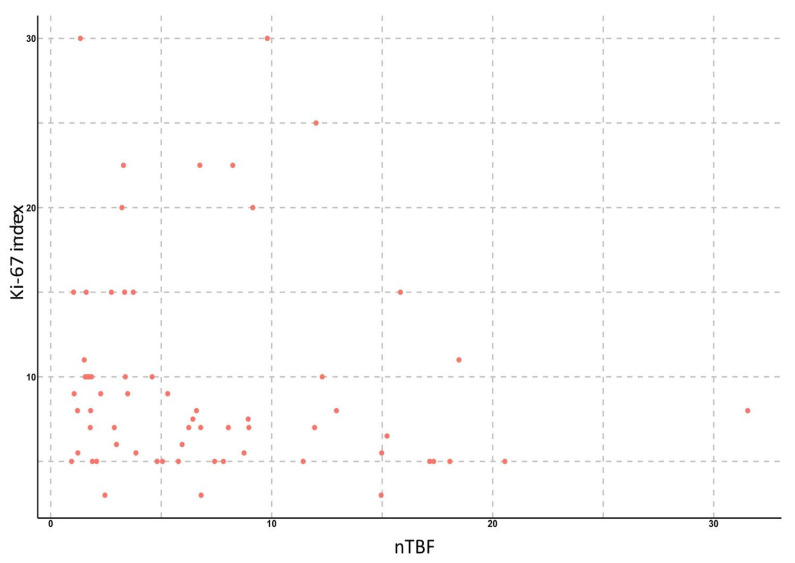
Scatterplot demonstrating the relationship between maximum normalized TBF in gliomas of various grades and Ki-67 index.

To study the relationship between the Ki-67 proliferative activity index and TBF, we analyzed ASL data in 65 patients with gliomas of various grades. We found significant correlations between TBF, as well as nTBF, and the Ki-67 index: the Spearman correlation coefficient for the maximum values of TBF was 0.63 (*p* < 0.001), 95% confidence interval ranged from 0.47 to 0.75. For normalized values, the correlation coefficient was 0.61 (*p* < 0.001), 95% confidence interval ranged from 0.42 to 0.74. In subgroup analysis (by grade or IDH1-status), no significant correlation between TBF and Ki-67 was detected (Figure 6 and Figure 7).

Thus, we could conclude that the higher the maximum TBF is, the more aggressive growth tumor demonstrates.

## 4. Discussion

Our study revealed significant difference in blood flow measured by the PCASL method in groups of gliomas with different IDH1 statuses. At the same time, wildtype gliomas showed higher TBF values compared to mutant tumors in the analysis of all glioma grades (Grade 1–4), HGG (Grade 3–4), and glioblastomas (Grade 4). ROC analysis demonstrated this technique to be efficient in the diagnosis of wildtype and mutant gliomas. IDH-mutant tumors tend to have downregulated hypoxia-dependent signaling and especially vasculo- and angiogenesis, which results in lower perfusion [35].

We also found a significant correlation between TBF and the Ki-67 index, which could be explained by the fact that both tumor cell proliferation and neoangiogenesis are hypoxia-dependent processes [36].

In the available literature, there are few studies investigating the relationship between blood flow measured by ASL perfusion and IDH1 mutation in brain gliomas.

Liu et al. (2018) used PCASL technology in 56 patients [23]. However, only 15 patients with Grade 2–3 gliomas (8 IDH1-mutant and 7 with wildtype) were selected for subsequent analysis of blood flow in the subgroups of mutant and wildtype gliomas. In 25 glioblastoma patients included in this study, only wildtype gliomas were diagnosed. Due to a small number of patients and large heterogeneity of tumor IDH1 status, the authors did not compare blood flow in the wildtype and IDH1-mutant malignant gliomas. A comparison of blood flow in groups of Grade 2–3 gliomas with different IDH1 statuses revealed that wildtype gliomas had higher rates of TBF (*p* = 0.029) and nTBF (*p* = 0.065). ROC analysis was not performed due to a small sample of patients. The larger sample size in our study allowed us to detect high TBF values in wildtype gliomas in the analysis of HGG and glioblastomas regardless of grades.

Yamashita et al. (2015) analyzed blood flow and genetic status in 66 patients with glioblastomas using pulsed PASL technology (55 with wildtype gliomas and 11 with IDH1 mutation), while the perfusion study was performed only in 43 patients (34 in patients with wildtype glioblastomas and 9 with mutant forms of glioblastomas) [26]. The study also included patients with recurrent tumors in the study group (1 patient with wildtype glioblastoma, 6 patients with mutant forms of glioblastoma). The maximum and normalized tumor blood flow in the group of wildtype glioblastomas was significantly higher compared to the mutant forms (*p* < 0.05). The ROC analysis revealed the high sensitivity and specificity of this technique in the differential diagnosis of glioblastomas with different IDH1 statuses. AUC values were 0.850 for TBF and 0.873 for nTBF. Threshold values were 70.0 mL/100 g/min for TBF and 1.55 for nTBF. Since the PASL technology was used, and normalization of blood flow in the gray matter of the contralateral hemisphere was carried out, we were unable to compare the data obtained in the study with our results. The disadvantage of the reviewed work is the inclusion of patients with recurrent glioblastomas in the study group. Previous surgery, chemotherapy, or radiotherapy can lead to a decrease in neoangiogenesis and the appearance of artifacts from hemoglobin breakdown products, which in turn leads to a decrease in blood flow. Half of the patients with mutant glioblastomas in this study received prior treatment, which could have affected the results of the measurements, e.g., reducing TBF rates, predominantly in the IDH1-mutant glioblastoma group. Our research enrolled patients with brain gliomas who had been treatment-naïve prior to MRI.

In the study of N. Wang et al. (2019) TBF was assessed by PCASL in 52 patients with gliomas of various grades (Grade 1–1 (IDH−), Grade 2–15 (13 IDH+/2 IDH−), Grade 3–13 (9 IDH+/2 IDH−/2 NOS), Grade 4–24 (3 IDH+/21 IDH−). The authors measured mean TBF (the volume of interest included entire tumor without necrotic and cystic areas) and maximum TBF (small size ROI in the region of greatest perfusion) [17]. The maximum TBF in gliomas with different IDH1 status did not differ significantly. The mean TBF in wildtype gliomas was significantly higher than in mutant forms. Analysis of high-grade gliomas with different IDH1 statuses found significant differences in blood flow. We analyzed the maximum TBF and identified significant differences in the TBF in gliomas with different IDH1 statuses in Grade 1–4, Grade 3–4, and Grade 4 glioma groups.

Lu et al. (2021) did not find significant difference in blood flow between groups of gliomas with different IDH1 statuses [24]. The authors used the PCASL technology; ROI included the entire T2 FLAIR hyperintense zone.

In a study conducted by Brendle et al. (2018) the authors used PASL technology to measure blood flow in 40 patients with gliomas of different grades (22 with LGG and 18 with HGG) [22]. ROI included the entire solid part of the tumor without areas of necrosis and cystic elements. Blood flow in wildtype astrocytomas (excluding oligodendrogliomas) was significantly higher than in mutant forms of astrocytomas (*p* = 0.0066).

The relationship between TBF and Ki-67 index in patients with brain gliomas was studied only in several works. H. Fudaba et al. (2014) used PASL technology in 32 patients with gliomas of different grades [33]. The authors did not reveal any relationships between blood flow and the Ki-67 index. Kang et al. (2020) used PCASL technology in 27 patients with cerebral gliomas, but also did not reveal any relationship between these parameters [37].

Zeng et al. (2017) studied blood flow in 58 patients with brain gliomas with the PCASL method [34]. When data from all gliomas included in the analysis, regardless of the grade, was analyzed, no correlation was found. At the same time, the authors revealed a weak positive correlation between blood flow and Ki-67 in the group of gliomas with a low index of tumor proliferative activity and a more pronounced negative relationship in the group of gliomas with high proliferative activity.

In our study, all patients had only newly diagnosed tumors. Our results show that the blood flow in malignant wildtype gliomas (Grade 3–4) is significantly higher than in mutant tumors, and the identified blood flow threshold values could be easily introduced everyday practice. We also found that the blood flow in wildtype glioblastomas was significantly higher than in mutant forms.

We showed a high positive correlation between blood flow and Ki-67. Our data allow us to detect gliomas with possibly aggressive growth at the preoperative stage and thus to plan the timing of surgical intervention (urgent/elective) and patient consulting.

The limitation of our work is the heterogeneity of study groups: a small number of wildtype gliomas among Grade 2–3 tumors and a small number of mutant forms among Grade 4 gliomas.

In future studies, the prognostic value of TBF may be increased when ASL perfusion is used together with other MRI methods. The most probable combination is proton MR spectroscopy with D-2-hydroxyglutarate peak assessment. However, the latter method is technically more complicated, as it requires more refined MR scanner adjustment.

## 5. Conclusions

Wildtype gliomas show significantly higher ASL-TBF values compared to mutant forms (among Grade 1–4 tumors). Subgroup analysis of HGG (Grade 3–4) and glioblastomas (Grade 4) with different IDH1 statuses also demonstrated that wildtype gliomas from these subgroups had higher TBF values. The obtained results allow predicting potentially more aggressive wildtype gliomas at the preoperative stage. We also found a significant positive correlation between ASL-TBF and Ki-67 proliferative index.

## Figures and Tables

**Table 1:** Tumor distribution in the study group.

Histopathological Diagnosis	Grade, WHO	N	IDH
Gangliolioma	1	2	1 wildtype/1 NOS (not otherwise specified)
Pilocytic astrocytoma	1	2	2 NOS
Diffuse astrocytoma	2	26	4 wildtype/21 mutant/1 NOS
Oligodendroglioma	2	7	7 mutant
Pleomorphic xanthoastrocytoma	2	1	1 wildtype
Anaplastic astrocytoma	3	18	5 wildtype/10 mutant/3 NOS
Anaplastic oligodendroglioma	3	14	14 mutant
Anaplastic pleomorphic xanthoastrocytoma	3	2	2 wildtype
Glioblastoma	4	44	35 wildtype/6 mutant/3 NOS

**Table 2:** ROC-analysis data comparing maximum TBF and maximum normalized nTBF in wildtype and IDH1-mutant gliomas.

	TBF	nTBF
Area under the ROC Curve	0.783 (0.693–0.872)	0.791 (0.702–0.879)
Optimal Threshold	80.57	4.7
Specificity	75% (62.5–87.5)	77.1% (25–75)
Sensitivity	77.6% (65.5–87.9)	79.3% (68.9–89.7)

**Table 3:** ROC analysis data comparing maximum TBF and maximum normalized nTBF in groups of malignant wildtype and IDH1-mutant gliomas.

	TBF	nTBF
Area under the ROC Curve	0.719 (0.595–0.842)	0.729 (0.603–0.853)
Optimal Threshold	84.18	4.7
Specificity	83.3% (71.4–95.2)	88.1% (78.5–97.6)
Sensitivity	60% (43.3–76.7)	60% (40–76.7)

**Table 4:** ROC-analysis data comparing maximum TBF and maximum nTBF in groups of wildtype and IDH1-mutant glioblastomas.

	TBF	nTBF
Area under the ROC Curve	0.782 (0.55–1)	0.792 (0.57–1)
Optimal Threshold	80.57	4.6
Specificity	88.9% (77.8–97.2)	88.9% (77.8–97.2)
Sensitivity	66.7% (33.3–100)	66.7% (33.3–100)

## Data Availability

Not applicable.
